# What stays

**DOI:** 10.1017/S1478951525100709

**Published:** 2025-08-22

**Authors:** Miguel Julião

**Affiliations:** Departamento de Cuidados Paliativos, Equipa Comunitária de Suporte em Cuidados Paliativos PalCo, Amadora, Portugal

You held my hand
like it could keep me here –
as if touch
could argue with time.
The machines were loud,
but your silence was louder.
I saw the storm
behind your eyes,
and wanted to apologize
for dying.
But death doesn’t wait
for goodbyes
or perfect words.
It just slips in,
soft as breath,
hard as truth.
And still –
you kissed my forehead
like I was going somewhere
and you’d meet me later.
Love doesn’t end.
Not really.
It just changes form:
a scent on an old shirt,
a voice in the back of your mind,
a name you still whisper
when no one’s listening.
I’m not gone.
Not all the way.
What we had doesn’t die –
it just stays.

